# Progress in Surgery

**Published:** 1896-11-21

**Authors:** 


### PROGRESS IN SURGERY.

### RESPIRATORY SURGERY.

**Hypertrophic Pulmonary Osteo-Arthropathy.**—Several papers on this subject have lately been published, but, before referring to them, it may be as well to describe the differences which exist between this disease and acromegaly, with which it was at first confused. They are thus formulated by Marie in his original description of the disease ("Essays on Acromegaly," New Sydenham Society, 1891, p. 71): In hypertrophic pulmonary osteo-arthropathy the face does not present the long oval of acromegaly; the lower jaw is not enlarged, but there is marked thickening of the alveolar border of the upper jaw; the spinal curvature is lumbar and lower dorsal, whereas in acromegaly it is cervical and upper dorsal. Retro-sternal dulness, apparently due to increase in the size of the bronchial glands, is present in both. In acromegaly the carpo-metacarpal part of the hand is much increased in size, whereas in hypertrophic pulmonary osteo-arthropathy, with the exception of a little hypertrophy of the heads of the metacarpal bones, it is not increased in size, but in this disease the last phalanges are much more enlarged than the others, and the whole finger is increased in size to a greater degree than in acromegaly. The ends of the radius and ulna at the wrist also enlarge in hypertrophic pulmonary osteo-arthropathy, which they do not in acromegaly. The nails are much enlarged and longitudinally striated, and become curved, whereas in acromegaly they do not alter in size or shape. The changes in the foot and leg in hypertrophic pulmonary osteo-arthropathy are exactly analogous to those in the hand and lower forearm. In hypertrophic pulmonary osteo-arthropathy the increase of size depends on hypertrophy of the bones, but in acromegaly on an equal degree of hypertrophy of the bones and soft tissues. The shafts of the bones of the extremities and the ends of the bones forming the knee and ankle may enlarge in hypertrophic pulmonary osteo-arthropathy, and the joints tend to become somewhat flexed and stiff.

At the Pathological Society, Messrs. Thorburn and Westmacott demonstrated the post-mortem appearances of a case of hypertrophic pulmonary osteo-arthropathy (*B. M. J.*, May 30th, 1896). They had discovered a highly symmetrical and almost universal osteo-periostitis of the limb bones, with a trophic erosion of the cartilages of their joints; and in the viscera they found tuberculosis. Mr. Thorburn thought the case favoured the view that hypertrophic pulmonary osteo-arthropathy was the result of a diffused but very mild form of tuberculosis of bone. Mr. Godlee has recorded several cases of the disease (*Brit. Med. Journal*, July 18th, 1896, p. 117). He has not observed any difference between the changes in the fingers in pulmonary osteo-arthropathy and the ordinary clubbed fingers met with in chronic suppuration within the chest, and in heart disease, and skiagrams taken of the fingers did not show any changes in the phalanges. He considers that two conditions are included under the name hypertrophic pulmonary osteo-arthropathy—one, the form of osteo-arthritis, which is associated with chronic septic suppurations, such as gonorrhœa and leucorrhœa, in which the clubbing of the fingers is an independent complication; and the other, a condition of chronic periostitis starting in the neighbourhood of the joints, but ultimately involving the whole of the shafts of the bones. This form of the disease Godlee does not agree with Thorburn in thinking tuberculous, but suggests a possible connection with syphilis, which has not been inquired into. Mr. Godlee records one case in which, after the removal of a drainage tube from the pleural cavity (where it had been retained for four years), marked enlargement of the wrists and ankle and clubbing of the fingers disappeared. Cases of this disease are also recorded (*New York Med. Journal*, Jan. 11th, 1896) by Dr. Sydney Thayer, from the Johns Hopkins Hospital, and by Dr. Walters from the North London Consumption Hospital (*Brit. Med. Journal*, Feb. 8th, 1896).

**Pulmonary Abscess.** — Two cases are recorded (*Lancet*, August 22nd, 1896) by Dr. Frederick Smith and Mr. Treves, in which cavities in the lung were localised by the physical signs, and drained with success. The cause of the abscess seems very obscure in both cases. In one the patient, a man fifty years of age, was suddenly attacked by severe pain in the chest, and a few days later his breath became very offensive, and a cough with fœtid expectoration developed. At this time there were no physical signs of disease in the lung, but after two months (during which time the fœtid expectoration persisted) Dr. Smith discovered some dulness between the border of the right scapula and the vertebral spines opposite the middle of the scapula, but the auscultatory signs were not those of a cavity. A grooved needle failed to discover any pus in this area, but Dr. Smith felt so certain that a cavity existed that Mr. Treves explored the lung. A flap of the tissues of the chest wall was raised, portions of the ribs removed, and an incision made into the lung, thus evacuating offensive pus from a cavity the size of a walnut. The abscess cavity was scraped and stuffed with iodoform gauze, and subsequently irrigated daily. It rapidly closed, but on return of pulmonary symptoms the affected area of the lung was again explored, but the old abscess cavity was found to be entirely obliterated. In the second case fœtid smell with the breath was the first symptom; there was only slight cough. No mention is made of fœtid expectoration. The physical signs of cavity in the lung were by no means distinctive, but one was discovered on operation. The lung was exposed in the same way as in the preceding case, but the lung had to be incised to the depth of an inch before the cavity was discovered. It was the size of a cricket ball, and was dealt with in the same way as in the first case, and with a satisfactory result. Mr. Treves did not suture the layers of pleura together before opening the abscess, but shut off the general pleural cavity from the area of lung to be explored by iodoform gauze packing. The abscess was kept plugged with iodoform gauze for several days. Mr. Treves considers that by dealing with the abscess in this way, it is unnecessary to suture the two layers of pleura. He draws attention to the fact that there was very little bleeding from the incision in the lung, though it extended to the depth of an inch.

Dr. W. A. Wills records (June 6th, 1896) a case in which a cavity in the lung was diagnosed, and an attempt made to drain it, but at the necropsy only general dilatation of the bronchial tubes, with consolidation of the surrounding lung, was discovered. The physical signs of a cavity were most marked at the upper part of the right lung, and Mr. Pollard resected a portion of the third rib in the axilla, and discovering adhesion of the two layers of pleura at this spot, he punctured the upper part of the lung with an exploring syringe in several places, but no pus was withdrawn. A few days after the operation the temperature began to rise, and the boy died. There was a history of cough and loss of flesh for two years, with latterly offensive expectoration. As the physical signs were limited to one lung, phthisis could be excluded in making the diagnosis. The whole of the right lung was found at the post-mortem to be quite solid, and the bronchial tubes were all dilated, and had thick, tough walls, but were not sacculated. There was no history of the entrance of any foreign body into the bronchus, nor was any found post-mortem; but it seems possible that a foreign body might have entered the bronchus without any remembrance of the occurrence on the part of the child, who was only seven years of age when the symptoms commenced, and that the foreign substance might have been expelled in coughing. The case very closely resembles one referred to in the "Surgical Progress" in THE HOSPITAL, in which, after the inhalation of a foreign body, the physical signs of a large cavity at the right apex developed, together with profuse and offensive purulent expectoration, and an attempt was made to reach the cavity, but the girl died from the entrance of pus into both bronchi during the anæsthesia. At the post-mortem only consolidation of the lung, with general bronchial dilatation and quite a small cavity, were discovered. Reference is made by Dr. Wills to a case of Dr. Goodhart's, in which the inhalation of a pea into the bronchus led to unilateral general bronchiectasis and localised empyæma, and to several cases in which bronchiectatic cavities—not due to impaction of a foreign body—have been diagnosed and drained. The great difficulty which might exist in finding such a cavity, deeply situated in the lung, and the risks of hæmorrhage which attend such exploration, are well exemplified in one case recorded by Dr. Theodore Williams and Mr. Godlee (*Trans. Royal Medico-Chirurg. Society*, vol. lxix.).

A case of abscess of the chest wall, associated with fracture of the first rib, is recorded by Dr. Jameson Johnston, of Dublin (*Dublin Journal of Medical Science*, Sep., 1896, p. 191). Fracture of the first rib alone is an excessively rare form of injury, and in this case it was supposed to be due to direct violence, which has never before been known. An abscess formed over the front of the chest just below the clavicle, and a communication with the lung was established, so that the pus was expectorated. After incision into and drainage of the abscess cavity, the expectoration of pus ceased, and the cavity closed. A fracture of the first rib was discovered by the finger of the operator when exploring the interior of the abscess cavity. Reference is made to a case of fracture of the first rib, recorded by Mr. Morton, in which an abscess formed at the root of the rib, and empyæma followed.

**Double Empyæma.**—A case of this nature is recorded by Mr. Huntington (*Lancet*, Aug. 15th, 1896, p. 459). On one side the empyæma was treated by incision and drainage, and on the other by aspiration. When the incision into the empyæma first operated on had closed, the other empyæma was also opened and drained, with a satisfactory result.

**Hydated Cyst of the Pleural Cavity.**—A case is recorded by Dr. Luff (*Lancet*, April, 25th, 1896) of an enormous hydated cyst, which filled the upper part of the adominal and lower part of the thoracic cavity, and was supposed to be situated in the liver, but on operation it was discovered to lie above the diaphragm, the left lobe of the liver being much compressed. There was evidence to show that the cyst had originated in connexion with the liver, but had grown upwards into the pleural cavity. The cyst was evacuated of daughter cysts and drained with success.

**Serous Expectoration after Paracentesis for Serous Effusion.**—Dr. Samuel West (*Lancet*, April, 18th, 1896) calls attention to the rare presence of this condition. The expectoration may occur after aspiration or syphon evacuation of the fluid, and varies considerably in quantity. It may come on during paracentesis or some hours later. Dyspnœa to a serious extent might be associated with it. The difference in the chemical character of the fluid in the effusion and that expectorated showed that it was not due to a puncture of lung with the aspirator trochar. It was considered, most probably due to œdema of the lung associated with pulmonary thrombosis.

